# Application of failure mode and effects analysis (FMEA) to pretreatment phases in tomotherapy

**DOI:** 10.1120/jacmp.v14i5.4329

**Published:** 2013-09-06

**Authors:** Sara Broggi, Marie Claire Cantone, Anna Chiara, Nadia Di Muzio, Barbara Longobardi, Paola Mangili, Ivan Veronese

**Affiliations:** ^1^ Servizio di Fisica Sanitaria Ospedale San Raffaele Milano Italy; ^2^ Dipartimento di Fisica Università degli Studi di Milano Milano Italy; ^3^ Servizio di Radioterapia Ospedale San Raffaele Milano Italy

**Keywords:** tomotherapy, failure mode and effects analysis, risk assessment, patient safety

## Abstract

The aim of this paper was the application of the failure mode and effects analysis (FMEA) approach to assess the risks for patients undergoing radiotherapy treatments performed by means of a helical tomotherapy unit. FMEA was applied to the preplanning imaging, volume determination, and treatment planning stages of the tomotherapy process and consisted of three steps: 1) identification of the involved subprocesses; 2) identification and ranking of the potential failure modes, together with their causes and effects, using the risk probability number (RPN) scoring system; and 3) identification of additional safety measures to be proposed for process quality and safety improvement. RPN upper threshold for little concern of risk was set at 125. A total of 74 failure modes were identified: 38 in the stage of preplanning imaging and volume determination, and 36 in the stage of planning. The threshold of 125 for RPN was exceeded in four cases: one case only in the phase of preplanning imaging and volume determination, and three cases in the stage of planning. The most critical failures appeared related to (i) the wrong or missing definition and contouring of the overlapping regions, (ii) the wrong assignment of the overlap priority to each anatomical structure, (iii) the wrong choice of the computed tomography calibration curve for dose calculation, and (iv) the wrong (or not performed) choice of the number of fractions in the planning station. On the basis of these findings, in addition to the safety strategies already adopted in the clinical practice, novel solutions have been proposed for mitigating the risk of these failures and to increase patient safety.

PACS number: 87.55.Qr

## I. INTRODUCTION

The benefits of ionizing radiation in medicine are well accepted even though the risks, coupled with their use, cannot be entirely eliminated. While the diagnostic use of radiation requires suitable methodologies to minimize the dose without impairing the diagnostic quality,^(1)^ the optimization in radiotherapy must be achieved by maintaining sufficiently high doses to irradiated tumors and protecting, at the same time, the healthy tissues to the largest extent possible.^(2)^

In modern radiotherapy (RT), much effort is being invested to improve the conformity of dose distribution, as well as to integrate imaging techniques for tumor tracking and correction of inter‐ and intrafraction variations.^(^ ^3^ ^,^ ^4^ ^)^ Intensity‐modulated radiation therapy (IMRT) is becoming the standard technique for achieving highly conformal irradiation volumes in many RT treatments through the intersection of numerous beamlets. Most commercial IMRT systems evolved from conventional linear accelerators (linacs) equipped with multileaf collimators (MLCs). A specifically designed IMRT machine, which also integrates a highly image‐guided system, is the helical tomotherapy. Indeed, a tomotherapy accelerator basically combines the main features of a linear accelerator and a megavoltage computed tomography (MVCT) scanner.^(5)^

A common aspect of all the new technologies and methodologies introduced in the modern RT is the level of complexity, evidently much higher than the recent past (i.e., before IMRT became a major radiotherapy treatment modality). The increased complexity related to the technological and process changes in RT places new demands on quality assurance (QA) programs, as well as innovative instruments and detectors for beam characterization and checks.^(^ ^6^ ^,^ ^7^, ^8^ ^)^ Moreover, new approaches to safety are required, since complexity may also increase the sensitivity to uncertainties and risk for accidental exposures. Examples of radiotherapy‐related errors are unfortunately not uncommon, even in the countries with the highest level of health‐care resources.^(9)^

In order to fully assess and manage the risks of accidental exposures deriving from the use of innovative radiotherapy methodologies, prospective approaches, widely applied in high‐risk industry, should be implemented to determine all the elements that could go wrong and identify, a priori, all the potential hazards that might occur during a radiotherapy treatment. ^(10)^ Prospective methods for risk analysis and patient safety improvement have been recently applied in various modern radiotherapy methodologies.^11^, ^12^, ^13^, ^14^, ^15^, ^16^, ^17^ However, as far as authors know, no studies specifically dedicated to tomotherapy are available in the literature.

The aim of this paper was the application of the failure mode and effects analysis (FMEA) approach to assess the risks for patients during the pretreatment phases in tomotherapy. The applied procedure included the definition of the processes and fault trees, the identification and scoring of each potential failure mode, and finally the suggestion of additional safety measures for process improvement and risk mitigation.

## II. MATERIALS AND METHODS

FMEA is a prospective risk analysis approach routinely employed in several manufacturing sectors, as well as in aviation. Recently FMEA was identified as a powerful tool in modern radiation oncology by the Task Group 100 of the American Association of Physicists in Medicine (AAPM).^(17)^ The use of the FMEA approach was also recommended by the International Commission on Radiological Protection (ICRP) as a resource for improving the safety of patients undergoing modern radiation therapy treatments.^1(10)^

In this study, FMEA was applied to identify all the subprocesses involved in the stages of (i) preplanning imaging and volume determination, and of (ii) treatment planning, characterizing a RT process performed by using a helical tomotherapy unit (HTU). Afterwards, the potential failure modes (i.e., what could go wrong), together with their causes and effects, were identified and ranked in order of importance. Three indexes were assigned for each failure mode: the occurrence rating (O), the severity rating (S), and the detectability rating (D). A ten‐point scale was used to score each category, ten being the number indicating the most severe, most frequent, and least detectable failure mode, respectively. The ranking scales proposed by Ford et al.^(15)^ were adopted as guidelines. Finally, the risk probability number (RPN) was calculated as the product of the three attributes: RPN = O × S × D. As already applied in previous FMEA studies in ${\text{RT}}_{,}^{\left(11‐13,15‐17\right)}$ the value RPN = 125 was considered as a threshold below which the risk can be considered acceptable. However, it must be pointed out that this value, derived from industry, still remains somehow arbitrary when applied to RT and deserves further investigation.

The analysis was carried out by a working group (WG) composed by five people working at the San Raffaele Scientific Institute (three medical physicists and two radiation oncologists), and by two additional external physicists with experience and competences in radiation protection and in risk management strategies for radiotherapy.

The delineation of the process trees and the identification of the potential failure modes, and causes and effects, were initially carried out through small group meetings. Afterwards, various collegial discussions were organized to revise the results the groups, and to identify and examine the additional safety measures for the risk mitigation. The risk attributes associated to each failure mode were initially conceived by each member of the WG in “blind mode”, then collectively revised during a dedicated plenary session to reach general consensus. The O, S, and D indexes for each failure mode were assigned by taking into account the current QA program and protocols developed at San Raffaele Scientific Institute and the safety measures already implemented.^(5)^

Indeed, San Raffaele Scientific Institute was assumed as a reference for the detailed definition of the process tree and the estimation of RPN numbers. The Institute is equipped with three HTUs: the first one (TT1, Hi‐Art II) installed in August 2004, a second (TT2, Hi‐Art II) installed in May 2007, and a third one (TT3, Tomotherapy HD) installed in March 2012 (TomoTherapy Inc., Madison, WI). Currently, only the two newer HTUs are operating; the first unit is at the moment off and will be replaced with a new one. The two operating HTUs (TT2 and TT3) are twinned each other; the same beam model is applied and each unit is characterized by a proper MLC with specific mechanical characteristics and specific output factors. Each HTU is connected with one specific operator station (OS) and with its specific tomotherapy planning station (ver. 4.2.1.2), a dedicated treatment planning system (TPS) equipped with an inverse planning algorithm able to optimize and calculate IMRT treatment plans. A planning transfer station (PTS) is also connected to each unit in order to allow the patient to transfer from different treatment machines. Each HTU, operator station, planning station, and PTS are connected into its own database.

In addition to the HTUs, the radiotherapy department is equipped with two conventional linacs (one Clinac 2100CD and one Clinac iX; Varian Medical Systems, Palo Alto, CA) able to deliver IMRT and volumetric‐modulated arc treatments (RapidArc). A simulator Acuity (Varian) and a dedicated CT GE HiSpeed (GE Healthcare, Waukesha, WI) are also installed in the radiotherapy department.

The staff of the Radiotherapy Department includes ten radiation oncologists and 18 radiographers. The Medical Physics Department includes nine medical physicists, one radiographer, and four technicians. Around 200 treatments were performed every year on each HTU.

## III. RESULTS & DISCUSSION

**Figure 1:**
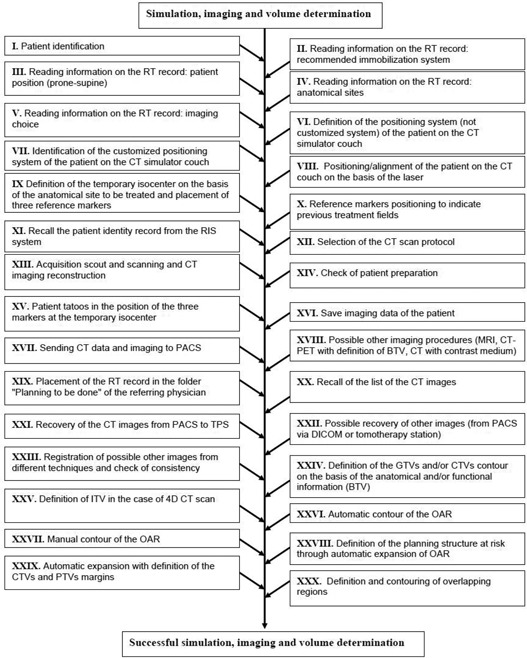
Subprocesses of the preplanning imaging and volume determination stage in tomotherapy.

**Figure 2:**
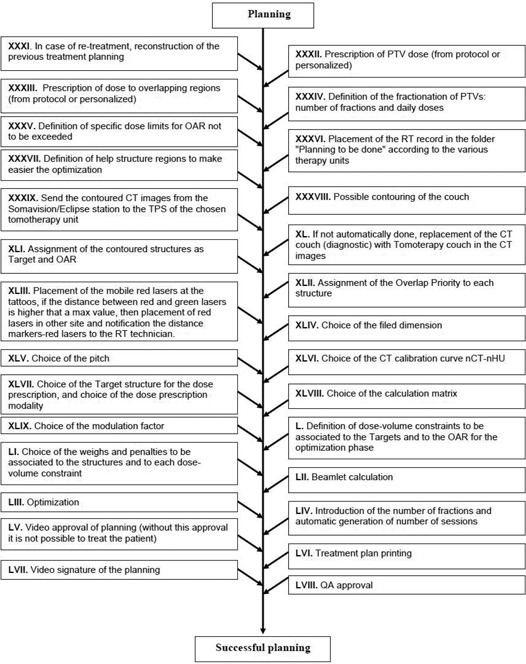
Subprocesses of the planning stage in tomotherapy.

The process tree of the preplanning imaging and volume determination stage is shown in Fig. 1; the process tree of the planning stage is reported in Fig. 2. Globally, 58 subprocesses were identified, starting from the patient identification up to the QA approval of the treatment plan. All the subprocesses were judged to be potentially prone to one or more failure modes.

A total of 74 failure modes were identified: 38 in the stage of preplanning, imaging, and volume determination, and 36 in the stage of planning. Fifty‐three failures (i.e., 72% of the cases) were considered of little concern in view of the RPN value lower or equal to 80. These failures are summarized in 1, 2 in a condensed form. A recurring minor failure in Table 1 was the improper imaging. It may occur in many subprocesses and, in most of cases, can be easily detected during the target/OAR contouring phase or simply during the upload and the visualization of the CT images. Typical examples are short CT slices acquisition, CT acquisition without an adequate patient preparation for special protocols (e.g., full/empty rectum, full/empty bladder), CT acquisition without an optimal immobilization system). In these cases the effect on patient safety is of little concern, consisting of additional CT exposure and consequent delay in the successive procedures. These same effects may occur when imaging data are lost, incomplete or corrupted during data transfer and saving procedures.

In other cases, improper imaging could be more difficult to detect during the planning stage, as attested by severe incidents reported in the recent past.^(9)^ In particular, a possible failure is the reversal of images as a consequence of incorrect positioning of the patient and/or incorrect selection of the CT scan protocol (i.e., “head first” vs. “feet first”).^(18)^ However, in tomotherapy, thanks to the check of the patient position performed just before the treatment by means of the MVCT scanner, the detectability of this failure can be considered sufficiently high to make the overall risk acceptable. Furthermore, the MVCT pre‐treatment imaging permits identification of possible discrepancies between the position taken by the patient during the preplanning imaging and that required for the treatment (e.g., arms up/down).

Possible minor failures in the planning stage (Table 2) caused by lack of attention are the missing of prescription data such as dose to planned target volume (PTV) or to overlapping regions, number of fractions, and dose limits to organs at risk (OAR). Since these data are strictly required by the medical physicist to start the treatment planning, their absence will only reflect in a delay of the process flow.

**Table 1:** Application of failure mode and effects analysis for the preplanning imaging and volume determination stage in tomotherapy. Failure modes having an assigned RPN ≤ 80 are listed

	*Subprocess*	*Potential Failure Mode*
I	Patient identification	Imaging of the wrong patient at a different anatomical site
		Imaging of the wrong patient at the correct anatomical site
II	Reading information on the RT record: recommended immobilization system	Improper imaging
III	Reading information on the RT record: patient position (prone‐supine)	Improper imaging
IV	Reading information on the RT record: anatomical sites	Improper imaging
		Improper planning optimization
V	Reading information on the RT record: imaging choice	Improper imaging
VI	Definition of the positioning system (not customized system) of the patient on the CT simulator couch	Improper immobilization
VII	Identification of the customized positioning system of the patient on the CT simulator couch	Improper immobilization
VIII	Positioning/alignment of the patient on the CT couch on the basis of the laser	Improper imaging
IX	Definition of the temporary isocenter on the basis of the anatomical site to be treated and placement of 3 reference markers	Improper imaging due to impossibility to define red laser position
XI	Recall the patient identity record from the RIS system	Incorrect association CT imaging ‐ patient
XII	Selection of the CT scan protocol	Improper imaging
XIII	Acquisition scout and scanning and CT imaging reconstruction	Improper imaging ‐ wrong anatomical region
XIV	Check of patient preparation	Improper imaging
XV	Patient tatoos in the position of the 3 markers at the temporary isocenter	Wrong isocenter
XVI	Save imaging data of the patient	Data loss
XVII	Sending CT data and imaging to PACS	Incomplete or corrupt data
		Inability to find the patient data, research on other nodes, and/or resending
XVIII	Possible other imaging procedures (MRI, CT‐PET with definition of BTV, CT with contrast medium)	Incomplete or corrupt data ‐ inability to recover the images
		Difficulty in the image fusion
XIX	Placement of the RT record in the folder “Planning to be done” of the referring physician	RT record loss
XX	Recall of the list of the CT images	CT images not found
XXI	Recovery of the CT images from PACS to TPS	Images associated to the wrong patient Impossibility to reconstruct previous treatments
XXII	Possible recovery of other images (from PACS via DICOM or tomotherapy station)	Incomplete or corrupt data
XXVI	Automatic contour of the OAR	Wrong OAR definition
XXVII	Manual contour of the OAR	Wrong OAR definition

Inadequate skill or lack of attention is also cause of other minor failures in the planning stage, such as the suboptimal choice of one or more parameters of the TPS (e.g., calculation matrix, field dimension, pitch, modulation factor, constrains). The possible effect of these failures was the delivery of a suboptimal treatment.

**Table 2:** Application of failure mode and effects analysis for the planning stage in tomotherapy. Failure modes having an assigned RPN < 80 are listed

	*Subprocess*	*Potential Failure Mode*
XXXI	In case of retreatment, reconstruction of the previous treatment planning	Wrong reconstruction Reconstruction not possible Reconstruction suboptimal
XXXII	Prescription of PTV dose (from protocol or personalized)	Missing prescription in the record
XXXIII	Prescription of dose to overlapping regions (from protocol or personalized)	Missing prescription in the record
XXXIV	Definition of the fractionation of PTVs: number of fractions and daily doses	Missing prescription in the record
XXXV	Definition of specific dose limits for OAR not to be exceeded	Missing prescription in the record
XXXVI	Placement of the RT record in the folder “Planning to be done” according to the various therapy units.	Missing record Planning for the wrong unit
XXXVII	Definition of help structure regions to make easier the optimization	Incorrect help structure region definition
XXXIX	Send the contoured CT images from the Somavision/Eclipse station to the TPS of the chosen tomotherapy unit	Wrong data sent
XLI	Assignment of the contoured structures as target and OAR	Wrong assignment
XLIII	Placement of the mobile red lasers at the tattoos, if the distance between red and green lasers is higher that a max value, then placement of red lasers in other site and notification the distance markers/red lasers to the RT technician.	Wrong positioning
XLIV	Choice of the filed dimension	Suboptimal choice
XLV	Choice of the pitch	Suboptimal choice
XLVII	Choice of the target structure for the dose prescription, and choice of the dose prescription modality	Wrong choice
XLIX	Choice of the modulation factor	Suboptimal choice
L	Definition of dose‐volume constraints to be associated to the targets and to the OAR for the optimization phase	Suboptimal choice
LI	Choice of the weights and penalties to be associated to the structures and to each dose‐volume constraint	Suboptimal choice
LII	Beamlet calculation	Beamlet recalculation
LIII	Optimization	Incomplete optimization
LV	Video approval of planning (without this approval it is not possible to treat the patient)	Approval missing
LVI	Treatment plan printing	Printed copy of the treatment plan missing
LVII	Video signature of the planning	Signature missing
LVIII	QA approval	QA approval missing

Twenty‐one failure modes (28% of cases) were characterized by a RPN score higher than 80. Ten of them, shown in Table 3, were identified in the stage of preplanning imaging and volume determination. The remaining 11 failures, which may occur during the stage of plan‐ning, are shown in Table 4. The mean values of the risk indexes of Table 3 were O = 3.3 (range 3–4), S = 6.7 (range 5–8), and D = 4.8 (rage 3–6). Similarly, the mean values of the indexes of Table 4 were: O = 2.7 (range 2–4), S = 7.0 (range 5–8), and D = 6.2 (range 4–9). These data suggest that such events, on average, are infrequent (once or a few times a year) and not too difficult to detect (especially for the stage of preplanning imaging and volume determination), but potentially severe in terms of patient safety. The threshold of 125 for RPN was exceeded in four cases: one case only in the phase of preplanning imaging and volume determination, and three cases in the stage of planning. Actually, treatment planning is known to be one of the most critical phases within the whole RT process.^(9)^

**Table 3:** Application of failure mode and effects analysis for the preplanning imaging and volume determination stage in tomotherapy. Failure modes having an assigned RPN > 80 are reported

*Subprocess*	*No*	*Potential Failure Mode*	*Potential Causes of Failure*	*Potential Effects of Failure*	*O*	*S*	*D*	*RPN*
VIII Positioning/alignment of the patient on the CT couch on the basis of the laser	1	Wrong definition of the isocenter	Inadequate CT laser alignment	Systematic shift of the patient position	4	8	3	96
X Reference markers positioning to indicate previous treatment fields	2	Missing marker positions	Lack of attention or incomplete compilation of the RT record	Previous treatment not taken into consideration/suboptimal planning	4	6	3	96
XXIII Registration of possible other images from different techniques and check of consistency	3	Wrong registration	Consistency not verified	Wrong PTV and OAR definition	3	7	5	105
XXIV Definition of the GTVs and/or CTVs contour on the basis of the anatomical and/or functional information (BTV)	4	Wrong CTV definition	Lack of attention/inadequate skill	Wrong dose distribution	3	7	5	105
XXV Definition of ITV in the case of 4D CT acquisition	5	Incorrect ITV construction	Lack of attention/inadequate skill	Wrong PTV	3	6	5	90
XXVI Automatic contour of the OAR	6	Missing OAR definition	Lack of attention/inadequate skill	Unintended normal tissue irradiation	3	8	5	120
XXVII Manual contour of the OAR	7	Missing OAR definition	Lack of attention/inadequate skill	Unintended normal tissue irradiation	3	8	5	120
XXVIII Defnition of the planning structure at risk through automatic expansion of OAR	8	Wrong expansion	Lack of attention/inadequate skill	Unintended normal tissue irradiation	3	5	6	96
XXIX Automatic expansion with definition of the CTVs and PTVs margins	9	Wrong expansion	Lack of attention/inadequate skill	Unintended normal tissue irradiation	3	5	6	96
XXX Definition and contouring of overlapping regions	10	Wrong/missed definition	Lack of attention/inadequate skill or not detailed information on previous treatment	Unintended normal tissue irradiation or wrong dose distribution	4	7	5	140

**Table 4:** Application of failure mode and effects analysis for the planning stage in tomotherapy. Failure modes having an assigned RPN > 80 are reported

*Subprocess*	*No*	*Potential Failure Mode*	*Potential Causes of Failure*	*Potential Effects of Failure*	O	*S*	*D*	*RPN*
XXXII Prescription of PTV dose (from protocol or personalized)	11	Wrong prescription in the record	Lack of attention/inadequate skill	Wrong dose delivery	3	8	4	96
XXXIII Prescription of dose to overlapping regions (from protocol or personalized)	12	Wrong prescription in the record	Lack of attention/inadequate skill	Possible wrong dose distribution/wrong dose delivery	3	8	4	96
XXXIV Definition of the fractionation of PTVs: number of fractions and daily doses	13	Wrong prescription in the record	Lack of attention/inadequate skill	Wrong dose delivery	3	8	4	96
XXXV Definition of specific dose limits for OAR, not to be exceeded	14	Wrong prescription in the record	Lack of attention/inadequate skill	Unintended normal tissue irradiated	3	8	4	96
XXXVIII Possible contouring of the couch	15	Incorrect dose calculation	Incorrect positioning in the imaging	Wrong dose delivery	2	6	7	84
XL If not automatically done, replacement in the imaging of the CT couch (diagnostic) with tomoterapy couch	16	Incorrect dose calculation	Incorrect introduction of the couch position in the CT images	Wrong dose delivery	2	6	8	96
XLII Assignment of the Overlap Priority to each structure	17	Wrong assignment	Lack of attention/inadequate skill	Wrong dose distribution	4	7	7	196
XLVI Choice of the calibration curve nCT‐nHU	18	Wrong choice (kV‐MV)	Lack of attention/inadequate skill	Wrong dose calculation/wrong dose delivery	2	8	9	144
	19	Wrong choice (kV‐MV)	Lack of attention/inadequate skill	Wrong dose calculation/wrong dose delivery	2	5	9	90
XLVIII Choice of the calculation matrix	20	Suboptimal choice	Lack of attention/inadequate skill	Suboptimal treatment	3	5	6	90
LIV Introduction of the number of fractions and automatic generation of number of sessions	21	Wrong or not performed choice (erroneous use of the default value)	Lack of attention/inadequate skill	Wrong dose delivery	3	8	6	144

The failure mode with the highest risk (i.e., RPN = 140) within the stage of preplanning imaging and volume determination was the wrong or missing definition and contouring of the overlapping regions. Overlapping regions are, in general, defined to limit the prescription dose to some structures, both in case of overlapping between target volume and dose limit to organs at risk, and in case of retreatment. Typical examples of overlapping regions are the boundaries between PTV and chiasma and/or optical structures, PTV and anterior rectal wall, and PTV and duodenum and/or stomach. In tomotherapy, the overlapping regions of adjacent anatomical structures need to be defined and contoured, and a priority level has to be assigned during planning in order to achieve the correct dose distribution to the PTV and, at the same time, to spare the OAR.

If one or more overlapping regions are not properly defined or contoured by the radiation oncologist, the potential effect for the patient could be an unintended normal tissue irradiation or, more likely, a wrong dose distribution. The main cause of this failure was identified as a lack of attention or inadequate skill of the radiation oncologist in charge of defining and contouring the volumes of interest. Moreover, this failure might occur as a consequence of a lack of exhaustive clinical documentation about possible previous RT treatments. Indeed, the doses received by the various anatomical structures during previous irradiations have to be taken into account for defining the overlapping regions.

An additional safety measure was suggested by the working group for reducing the probability of occurrence of this failure, and therefore for mitigating the overall risk. It consisted of improving the RT record of the patient through the systematic introduction of a sheet to be flagged containing detailed information about the prescribed dose to target volumes, OARs, and overlapping regions contoured with relative dose constraints.

The most critical failure in the stage of planning (RPN = 196) was the wrong assignment of the overlap priority to each anatomical structure by the medical physicist. The TPS prompts the user to divide structures or regions of interest (ROI) into two groups: tumors and organs at risk. A ROI from the tumor group can overlap with a ROI in the OAR group, but the ROIs within the same group are not allowed to own common voxels. When two contours from the same group overlap with each other, the overlap priority setting governs to which structure the voxel belongs. The structure with the higher overlap priority will own the voxels in the overlap region, and the structure with lower overlap priority will lose these voxels for dose volume computations, optimization, and dose‐volume histograms (DVHs) in the overlapping mode visualization.^(19)^ Therefore, overlap priorities must be carefully selected since, in case of failure, wrong dose optimization can be generated with possible serious consequences for the patient. As for many other failures, this error was recognized as the result of a lack of attention or inadequate skill of the medical physicist in charge of planning. The probability of this failure going undetected was estimated to be relatively high if the final plan evaluation is based on only the DVH in the overlapping mode (i.e., the usual way to visualize the DVH during plan optimization). For this reason, the additional safety measure recommended by the WG consisted of a systematic final plan evaluation considering both the DVH in the overlapping mode and in the standard mode (i.e., the mode that provides the statistics of the entire volumes), regardless the defined overlapping priorities. It is interesting to note that the possibility to visualize and to print out the DVHs in the two different modes is an option of one of the latest TPS release.

A further critical failure identified in the planning stage (RPN = 144) was the wrong choice of the CT calibration curve. This failure might accordingly occur when more than one CT scanning system is available, each of them with the proper calibration curve. Since the dose calculation process by the TPS is based on the data of the CT calibration, the wrong choice of calibration curve could reflect in a wrong dose calculation and, possibly, in a wrong dose delivery. This failure could be particularly critical in tomotherapy since, in the case of using MVCT instead of kVCT images, or vice‐versa, a significant variation in the calculated dose (i.e., of the order of 10%) is expected. Indeed, the relationship of MVCT number to electron density is different from that observed in common kVCT scanners as a consequence of the difference in physical interaction probabilities of photons within the two energy ranges.

This failure was considered extremely hard to detect (i.e., D = 9). Indeed, the information about the CT calibration curve chosen for dose calculation cannot be included in the printed copy of the treatment plan. Therefore, the correctness of this parameter cannot be checked during the plan evaluation before the final approval. On the basis of this analysis, the WG decided, as additional strategy, to suggest the vendor implement a new function in the TPS software, consisting of the possibility for the user to include the information on the CT calibration curve in the printed copy of the treatment plan.

The last major failure (with a RPN = 144) which might occur during the planning stage, was the wrong (or not performed) choice of the number of fractions composing the treatments. A default number of fractions equal to 30 is set in the TPS. This value must be changed by the planner according to the particular prescription. If the planner fails to perform this task, the consequences for the patient could be particularly severe, since a wrong daily dose could be delivered during the treatment. The additional safety measure recommended by the WG is to increase the probability of failure detection by the RT technician before the treatment start through the systematic check of the agreement between the number of fractions actually prescribed in the radiotherapy sheet and the number of procedures visualized on the screen of the tomotherapy control unit.

In addition to the failure modes with the highest overall risks, specific attention should be paid to the failures which could lead to severe injuries to the patients, independently of the RPN value. In this analysis, seven failure modes characterized by a severity index S = 8 and RPN <125 were identified (i.e., failures No. 1, 6 and 7 in Table 3 and Nos. 11–14 in Table 4). The wrong definition of the isocenter during the positioning of the patient on the CT couch, even though potentially dangerous, appeared easily detectable, since the laser alignment is daily checked through the use of the MVCT scanner. No further safety measures are, therefore, required.

The risk of missing OAR definitions can be simply mitigated through the implementation of the safety measures formerly proposed. In particular, the suggested improvement of the RT record could significantly reduce the number of occurrences of these failures.

Finally, additional safety measures are required to deal with the remaining failures — the wrong prescription of dose and/or dose limits. Systematic double‐check of these data by the radiation therapy staff, and/or the definition of reference protocols, could be an adequate solution increasing the detection rating of these failures.

The failure modes shown in 1, 2, 3, 4 were identified by analyzing the typical sequence of events and procedures that characterize the standard RT process. However, in clinical practice, some deviations from the standard process flow might occur as a consequence of specific medical or organizational needs. Unfortunately, such process changes cannot be considered free from additional potential failures. In particular, three events which could affect the patient safety were identified by the working group and are briefly described below.

1. When the prescribed dose to the PTV is high, it may occur that the HTU is not able to deliver the entire dose in a single fraction; the physicist has to duplicate the number of fractions and write in the radiotherapy sheet to deliver two fractions every daily treatment session. If the indication is not clearly reported, this can result in an uncorrected daily dose delivery to the patient.

2. Sometimes, it could happen that the radiation oncologist decides to deliver more fractions than planned (e.g., if the treatment needs to be interrupted in a case where the patient experienced severe side effects). The TPS does not allow the physicist to add treatment fractions without replanning and recalculating the plan, but only those QA fractions on the operator station. So the patient can be treated, but the final treatment report does not record the added fractions and only the radiotherapy sheet can attest the real delivered dose.

3. As previously reported, at San Raffaele Scientific Institute two HTUs are currently operating. The two machines are twinned in order to have the possibility to transfer the patient from one unit to the other one (in a case where one machine needs maintenance). In such a case, the patient should be immediately cancelled from the original unit, transferred to the second one, treated, deleted from the TPS of the second unit, and finally transferred again to the original unit. In fact, this procedure would permit the tracking of the treatments actually done by the patient on the two units. However, it must be considered that every transfer entails a new final dose calculation (taking into account the specific output factors and MLC mechanical characteristics of each unit); moreover, each of these operations takes a lot of time to be completed. So, usually the patient record remains simultaneously on the two TPS for few days and, in this way, the only possibility to check the number of fractions actually delivered is through the radiotherapy sheet of the patient.

In all these circumstances, more flexibility of the system should let the operator to modify the schedule of the treatment so as to avoid communication errors that can induce an incorrect dose to patients. One solution could be the implementation of a record and verify system; a session, daily, and total dose accumulation point could also be a safety issue.

In addition to the safety measures specifically related to tomotherapy proposed by the WG, the results of this analysis confirmed the soundness of the general lessons and recommendations provided by ICRP for preventing accidental exposures from new external beam radiation therapy technologies.^(10)^ Indeed, during the identification of the causes of failures and the estimation of their occurrence probability, it came out that the competence and skill of the various professional figures involved in the RT process, as well as the work environment, play an important role both for the quality of the treatment and for patient safety. Regular updating of the knowledge and maintenance of the skills of the personnel can, therefore, represent an efficient instrument for patient safety improvement. In particular, the development of specific professional training schemes on the functionalities and limits of the various systems and software used in the RT process, as well as on the procedures and protocols, can be considered a general action for reducing the frequency of failures and, consequently, the overall risk of accidents in tomotherapy. Furthermore, excessive workload should be avoided, and a suitable work environment that encourages working with awareness, facilitates concentration, and avoids distraction should be provided.

Finally, as in other modern RT techniques, in tomotherapy *in vivo* dosimetry could provide a reliable way of detecting serious anomalies potentially leading to substantial overdoses or underdoses to the patient.

## IV. CONCLUSIONS

The application of FMEA to the preplanning imaging, volume determination, and treatment planning stages in tomotherapy led to the identification and deep investigation of various failure modes. The assignment of a score assessing the potential risk for each event permitted a ranking of these failure modes in order of importance and the ability to define priorities for risk mitigation, with the aim of optimizing quality management workflow. In addition to the safety strategies already adopted in the clinical practice, novel solutions have been proposed to increase patient safety.

Although this study was carried out considering the specific processes implemented at San Raffaele Scientific Institute, the proposed methodology, as well as likely most of the findings, can be generalized and made useful to other RT centers equipped with HTU.

On the basis of the results obtained in this study and of the experience accrued by the WG,^(20)^ further stages of the RT process, such as treatment delivery and treatment monitoring and verification, will be analyzed by means of the FMEA approach in the near future.
